# Brain structure abnormalities in adolescent girls with conduct disorder

**DOI:** 10.1111/j.1469-7610.2012.02617.x

**Published:** 2012-10-22

**Authors:** Graeme Fairchild, Cindy C Hagan, Nicholas D Walsh, Luca Passamonti, Andrew J Calder, Ian M Goodyer

**Affiliations:** 1 Department of Psychiatry, University of CambridgeCambridge, UK; 2 School of Psychology, University of SouthamptonSouthampton, UK; 3 Medical Research Council Cognition and Brain Sciences UnitCambridge, UK; 4 Consiglio Nazionale delle Ricerche, Unità di Ricerca NeuroimmaginiCatanzaro, Italy

**Keywords:** Conduct disorder, callous-unemotional traits, voxel-based morphometry, anterior insula, amygdala, sex differences

## Abstract

**Background:**

Conduct disorder (CD) in female adolescents is associated with a range of negative outcomes, including teenage pregnancy and antisocial personality disorder. Although recent studies have documented changes in brain structure and function in male adolescents with CD, there have been no neuroimaging studies of female adolescents with CD. Our primary objective was to investigate whether female adolescents with CD show changes in grey matter volume. Our secondary aim was to assess for sex differences in the relationship between CD and brain structure.

**Methods:**

Female adolescents with CD (*n* = 22) and healthy control participants matched in age, performance IQ and handedness (*n* = 20) underwent structural magnetic resonance imaging. Group comparisons of grey matter volume were performed using voxel-based morphometry. We also tested for sex differences using archive data obtained from male CD and control participants.

**Results:**

Female adolescents with CD showed reduced bilateral anterior insula and right striatal grey matter volumes compared with healthy controls. Aggressive CD symptoms were negatively correlated with right dorsolateral prefrontal cortex volume, whereas callous-unemotional traits were positively correlated with bilateral orbitofrontal cortex volume. The sex differences analyses revealed a main effect of diagnosis on right amygdala volume (reflecting reduced amygdala volume in the combined CD group relative to controls) and sex-by-diagnosis interactions in bilateral anterior insula.

**Conclusions:**

We observed structural abnormalities in brain regions involved in emotion processing, reward and empathy in female adolescents with CD, which broadly overlap with those reported in previous studies of CD in male adolescents.

## Introduction

There are marked sex differences in the prevalence of antisocial behaviour, with male adolescents being more likely than female adolescents to commit violent crimes or meet diagnostic criteria for conduct disorder (Moffitt, Caspi, Rutter, & Silva, 2001). However, rates of violent crime and conduct disorder/oppositional defiant disorder (CD/ODD) diagnoses have risen significantly amongst adolescent females in the UK and USA in recent years (Collishaw, Maughan, Goodman, & Pickles, 2004; Federal Bureau of Investigation, 2006; Youth Justice Board, 2009), making it increasingly important to study this population. In female adolescents, CD is associated with a range of negative outcomes, including teenage pregnancy, antisocial personality disorder and mental and physical health problems in adulthood (Bardone et al., 1998; Odgers et al., 2008; Pajer, 1998).

Accumulating evidence suggests that neurobiological factors may be involved in the aetiology of CD. However, almost all previous structural and functional neuroimaging studies of CD have been restricted to male adolescents alone. This work has documented structural abnormalities in the amygdala, insula and orbitofrontal cortex (Fairchild et al., 2011; Huebner et al., 2008; Sterzer, Stadler, Poustka, & Kleinschmidt, 2007) and reduced amygdala activation during facial emotion processing in male adolescents with CD (Passamonti et al., 2010) or male children with conduct problems and callous-unemotional (CU) traits (Jones, Laurens, Herba, Barker, & Viding, 2009; Marsh et al., 2008). Whether CD is associated with a similar neurobiological profile when it occurs in female adolescents remains currently unknown. Although there is some evidence for sex differences in the relationship between psychophysiological measures and aggressive behaviour (Beauchaine, Hong, & Marsh, 2008) or psychopathic traits (Isen et al., 2010), there are also reasons to suspect that male adolescents and female adolescents with CD may show similar abnormalities in brain structure and function. First, both male adolescents and female adolescents with CD show reduced basal cortisol levels (McBurnett, Lahey, Rathouz, & Loeber, 2000; Pajer, Gardner, Rubin, Perel, & Neal, 2001), which is of interest because the amygdala is involved in regulating hypothalamic-pituitary-adrenal axis activity (Gunnar & Quevedo, 2007). Second, CD is associated with similar neuropsychological impairments in both sexes, including lower verbal IQ (Lynam, Moffitt, & Stouthamer-Loeber, 1993; Moffitt & Silva, 1988; Pajer et al., 2008), deficits in fear conditioning (Fairchild, Stobbe, van Goozen, Calder, & Goodyer, 2010; Fairchild, Van Goozen, Stollery, & Goodyer, 2008), startle responses (Fairchild et al., 2008, 2010) and recognition of facial expressions of anger and disgust (Fairchild, Van Goozen, Calder, Stollery, & Goodyer, 2009; Fairchild et al., 2010). Third, a recent study observed similar negative correlations between orbitofrontal cortex volume and antisocial personality disorder symptoms in male adults and female adults (Raine, Yang, Narr, & Toga, 2011).

Our primary objective was to test the hypothesis that female adolescents with CD would show abnormalities in brain structure, which overlap with those observed in male adolescents with CD, using voxel-based morphometry (VBM) to analyse structural magnetic resonance imaging (MRI) data. As all three previous structural MRI studies of male adolescents with CD (Fairchild et al., 2011; Huebner et al., 2008; Sterzer et al., 2007) reported reductions in amygdala grey matter volume, and two reported reduced anterior insula volume (Fairchild et al., 2011; Sterzer et al., 2007), we hypothesised that female adolescents with CD would show similar reductions in these regions. We also predicted that striatal volume would be decreased in female adolescents with CD, consistent with a previous study in male adolescents with CD (Fairchild et al., 2011). Finally, we expected female adolescents with CD to show reductions in orbitofrontal cortex volume given previous findings in adult males with antisocial personality disorder (Raine et al., 2011) or psychopathy (de Oliveira-Souza et al., 2008; Yang, Raine, Colletti, Toga, & Narr, 2010) and male children with CD and attention deficit/hyperactivity disorder (ADHD; Huebner et al., 2008).

We also assessed for dimensional relationships between brain structure and CU traits or CD symptoms. To evaluate the claim that CD with CU traits is associated with a qualitatively different neurological profile relative to CD without CU traits (Moffitt et al., 2008), we investigated whether individual differences in CU or psychopathic traits were related to grey matter volume. On the basis of previous studies in adult psychopaths (Glenn, Raine, Yaralian, & Yang, 2010; Glenn & Yang, 2012), and the two studies that investigated brain structure in children with conduct problems and CU traits (De Brito et al., 2009) or adolescents with CD and CU traits (Fairchild et al., 2011), we predicted that CU traits would be positively correlated with orbitofrontal cortex and striatal volumes, particularly in adolescent samples. These predictions were informed by a prior study showing that CU traits may be associated with delays in brain maturation, leading to increased grey matter volume or concentration in adolescents with CU traits (De Brito et al., 2009). We also assessed for relationships between CD symptoms and grey matter volume, predicting a negative correlation between CD symptoms and anterior insula volume (Fairchild et al., 2011).

Our final objective was to test for sex differences in the relationship between CD and brain structure by including data from male adolescents in the structural analyses. As CD is less common in females than males (Moffitt et al., 2001), it has been proposed that females may require a greater loading of neurobiological or psychosocial risk factors to develop antisocial behaviour (Cloninger, Christiansen, Reich, & Gottesman, 1978; Sigvardsson, Cloninger, Bohman, & von Knorring, 1982). This may be reflected in greater grey matter volume reductions in female adolescents with CD compared with male adolescents. Alternatively, sex differences in aggression and antisocial behaviour, which are most marked in late childhood (Côté, Vaillancourt, LeBlanc, Nagin, & Tremblay, 2007), may reflect sex differences in peer or parental socialisation of aggression (Keenan & Shaw, 1997; Maccoby, 1998). Direct comparisons of relationships between brain structure and CD in male adolescents and female adolescents may therefore be informative regarding the origins of sex differences in externalising psychopathology (Rutter, Caspi, & Moffitt, 2003).

## Method

### Participants

Twenty-two female adolescents with CD aged 14–20 years were recruited from schools, pupil referral units and the Cambridge Youth Offending Service. A healthy control group (HC; no history of CD/ODD and no current psychiatric illness) of 21 female adolescents, matched for age, handedness and performance IQ, was recruited from schools. All participants and their parents gave written informed consent to participate in the study, which was approved by the Suffolk NHS Research Ethics Committee. Exclusion criteria included full-scale IQ (FSIQ) <80, as estimated using the Wechsler Abbreviated Scale of Intelligence (Wechsler, 1999), and presence of pervasive developmental disorder (e.g. autism).

All participants were assessed for CD, ODD, ADHD, major depressive disorder (MDD), generalised anxiety disorder, obsessive compulsive disorder, post-traumatic stress disorder and substance dependence using the Schedule for affective disorders and Schizophrenia for School-Age Children-Present and Lifetime Version (Kaufman et al., 1997). Diagnostic interviews were carried out separately with participants and caregivers. Most (*n* = 17) of the female CD participants had adolescence-onset CD (i.e. onset of CD symptoms after age 10; American Psychiatric Association, 1994).

Self-reported CU and total psychopathic traits were assessed using the Callous-Unemotional (CU) subscale and total score of the Youth Psychopathic traits Inventory (YPI; Andershed, Kerr, Stattin, & Levander, 2002), respectively. The Adolescent Alcohol and Drug Involvement Scale measured alcohol and substance use (Moberg, 2000). Handedness was assessed using the Edinburgh Handedness Inventory (Oldfield, 1971). Finally, socioeconomic status (SES) was quantified using the ACORN geodemographic tool (http://www.caci.co.uk/acorn-classification.aspx).

## Neuroimaging methods

### Data acquisition

Structural MRI data were acquired using a 3-Tesla Siemens Tim Trio scanner at the MRC Cognition and Brain Sciences Unit, Cambridge, UK. We acquired T1-weighted 3D magnetisation-prepared rapid acquisition with gradient-echo images (voxel size = 1×1×1 mm, repetition time = 2250 ms, echo time = 2.99 ms, inversion time = 900 ms, flip angle = 9°). Total scanning time was 4 min 16 s.

### Image processing and analysis

VBM analysis was performed using SPM5 (Wellcome Department of Imaging Neuroscience, London, UK). Images were first inspected for scanner artefacts and then for gross neuroanatomical abnormalities, such as tumours or cysts, by a consultant radiologist (one control subject was excluded for this reason). The DARTEL toolbox in SPM5 was used to spatially segment the images, import them into Native Space, create a template from the merged images of the 42 subjects, and warp, modulate, normalise and smooth the individual results using an 8-mm full-width at half-maximum Gaussian kernel.

Following preprocessing, statistical analyses were performed in SPM5 using General Linear Models (GLMs) to permit quantification of group effects with total grey matter volume included as a covariate of no interest. Regression analyses examined whether CD symptoms (lifetime/ever, current, or aggressive symptoms) were correlated with grey matter volume, when considering the CD group alone. We also explored the effects of variation in psychopathic or CU traits in both the overall sample and the CD group alone. Two approaches for thresholding second-level maps were employed. We first performed a region of interest (ROI) analysis using a significance level of *p* < .05, Family-Wise Error (FWE) correction for multiple comparisons within the ROIs (i.e. small-volume correction; svc). Consistent with previous studies (Fairchild et al., 2011; Huebner et al., 2008; Passamonti et al., 2010; Sterzer et al., 2007), we defined the amygdala, anterior insula, striatum, anterior cingulate cortex and the superior, medial, middle and inferior subregions of the OFC as our ROIs using the atlas for automated anatomical labelling (Tzourio-Mazoyer et al., 2002). The anterior insula was defined by restricting the structural template for the insula to the region anterior to the anterior commissure plane (i.e. *y* > 0). We subsequently performed comparisons between groups at the whole-brain level (employing a statistical threshold of *p* ≤ .001 uncorrected, ≥10 contiguous voxels).

## Results

### Demographic and clinical data

Table 1 provides the results of group comparisons for the demographic and clinical measures. The groups were matched in age, performance IQ, handedness and ethnicity. However, the CD participants were of lower SES and had slightly lower FSIQ than controls.

**Table 1:** Demographic and clinical characteristics of the female participants

	Groups		
	
	HC (*n* = 20)	CD (*n* = 22)	Group comparisons (*p* values)
Age (years)	17.55 ± 0.67	17.23 ± 1.68	.42
Full-scale IQ	105.80 ± 9.52	99.77 ± 7.90	.03
Performance IQ	105.50 ± 11.49	101.54 ± 9.82	.24
Verbal IQ	107.90 ± 16.12	98.18 ± 16.32	.06
Handedness (R/L)	20/0	21/1	.52
Number of current DSM-IV diagnoses			
ADHD	0	2	.27
Substance Abuse	0	2	.27
Panic disorder	0	1	.52
Number of past DSM-IV diagnoses^a^			
ADHD	0	3	.13
MDD	3	10	.03
Substance Abuse	0	4	.07
PTSD	0	1	.52
Number of symptoms^b^			
Current CD	0.13 ± 0.34	2.73 ± 2.53	<.001
Lifetime CD	0.38 ± 0.62	7.59 ± 2.26	<.001
Aggressive CD	0.06 ± 0.25	2.64 ± 1.22	<.001
Current ADHD	1.60 ± 1.85	6.00 ± 3.34	<.001
Lifetime ADHD	1.95 ± 2.16	8.18 ± 3.70	<.001
YPI psychopathic traits	1.59 ± 0.31	2.07 ± 0.42	<.001
YPI CU traits subscale	0.52 ± 0.10	0.62 ± 0.13	.02
SES (ACORN)			
1 Wealthy achievers	9	4	
2 Urban prosperity	0	5	
3 Comfortably off	6	4	.04
4 Moderate means	0	1	
5 Hard-pressed	5	8	
Ethnicity			
Caucasian	20	21	.52
Nonwhite		1	

Data are presented as means ± standard deviation or number in each group. ADHD, attention deficit/hyperactivity disorder; CD, conduct disorder; CU, callous-unemotional; HC, healthy control; IQ, intelligence quotient; MDD, major depressive disorder; PTSD, post-traumatic stress disorder; SES, socioeconomic status; YPI, Youth Psychopathic traits Inventory.

a Numbers relate to those with a past diagnosis who were in remission at the time of the psychiatric assessment.

b For symptoms, current CD or ADHD indicates the number of symptoms present within the last 12 months; lifetime CD or ADHD indicates the number of symptoms that had been present within the participant’s lifetime, even if they were not currently present; aggressive CD symptoms included fighting, bullying, aggressive stealing, use of a weapon and physical cruelty.

Relative to controls, the CD group reported higher levels of self-reported total psychopathic traits and CU traits, and endorsed more CD and ADHD symptoms. CD participants were also more likely than controls to have had a lifetime diagnosis of MDD or ADHD.

### Structural MRI results: Female controls versus females with CD

Total grey matter volume did not differ between groups [*t*(40) = 0.49, *p* = .63].

**Figure 1:**
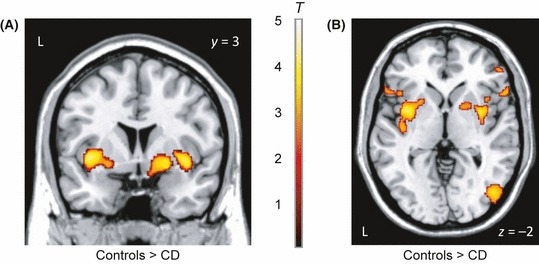
Bilateral anterior insula and right striatal grey matter volume was reduced in female adolescents with conduct disorder relative to healthy controls. Table 2 provides statistics and coordinates for these group differences. Panel A displays bilateral anterior insula and right striatal volume differences in coronal format, whereas panel B depicts the results in axial format. The colour bar, which ranges from red to white, represents *T* statistics. Both images are thresholded at *p* < .005, uncorrected, for display purposes

Relative to controls, the CD group showed reduced grey matter volume in bilateral anterior insula and right striatum (see Figure 1 and Table 2 for coordinates and statistics). A further cluster in right striatum (*p* = .06, svc), extending into ventral striatum, also showed a trend towards reduced volume. None of the ROIs were increased in volume in the CD group, compared with controls. A supplementary analysis comparing just the adolescence-onset CD subgroup (*n* = 17) with controls revealed similar reductions in bilateral anterior insula volume (Figure S1).

**Table 2:** Group differences in grey matter volume between the female conduct disorder group and healthy control subjects

				MNI coordinates		
				
Cerebral regions	Hemisphere	Local maxima, *z*	Number of significant voxels in cluster	*x*	*y*	*z*
HC > CD						
Anterior insula	L	4.00^a^	278	−39	3	−2
	R	3.82^a^	285	36	3	−3
Striatum	R	3.80^a^	Same cluster as above	34	2	−2
Striatum	R	3.64^b^	218	22	3	−8
Ventral striatum	R	3.32	Same cluster as above	15	9	−11
Orbitofrontal cortex	R	3.49	16	56	24	−11
	R	3.47	81	50	39	−17
Dorsolateral PFC	R	3.49	75	40	38	31
Precentral gyrus	L	3.44	25	−63	−16	37
Mid-occipital cortex	R	3.39	97	46	−75	0
Inferior frontal gyrus	R	3.37	49	62	21	6
Precuneus	L	3.36	31	−16	−70	60
CD > HC						
Middle temporal gyrus	R	3.57	87	54	−17	−23
Orbitofrontal cortex	R	3.44	27	15	32	−11
Precentral gyrus	R	3.37	55	35	−6	30

CD, conduct disorder; HC, healthy controls; MNI, Montreal Neurological Institute; PFC, prefrontal cortex.

a *p* < .05, Family-Wise Error (small-volume correction); ^b^*p* = .06, Family-Wise Error (small volume correction).

Grey matter reductions in all other regions met the criteria of *p* ≤ .001, uncorrected, for ≥10 contiguous voxels.

### Potential confounds

To investigate whether group differences in demographic, clinical, or personality variables contributed to our findings, we performed additional analyses, including these variables as covariates.

The group effects in bilateral anterior insula and right striatum remained significant (*p* < .05, svc) when controlling for FSIQ, SES, MDD and tobacco or alcohol use. When controlling for cannabis use, the bilateral anterior insula effects remained significant, but the striatal finding was no longer significant (*p* = .10, svc). The group effect in right anterior insula remained significant or showed a strong trend when controlling for either psychopathic traits (*p* = .05, svc) or CU traits (*p* < .05, svc). However, the left anterior insula and right striatal effects were not significant when controlling for these variables. Furthermore, the bilateral anterior insula and right striatal findings were reduced to trend effects (all *p* = .001, uncorrected) when controlling for either lifetime/ever or current ADHD symptoms. To explore this effect further, we excluded all subjects with a current or past ADHD diagnosis (*n* = 5) and repeated the analyses. The group effect in left anterior insula remained significant (*p* < .05, svc), although the effects in right anterior insula and striatum were no longer significant.

### Correlations with self-reported psychopathic or CU traits

We used regression analyses to investigate the relationship between psychopathic or CU traits and grey matter volume in the total female sample. Bilateral middle/superior OFC volume was positively correlated with self-reported CU traits (both *p* < .05, svc; Figure S2). These results remained significant when controlling for lifetime/ever CD or ADHD symptoms, suggesting that they were more strongly related to variation in CU traits than CD or ADHD symptoms. Bilateral anterior insula volume was negatively correlated with either self-reported psychopathic or CU traits (all *p* ≤ .05, svc), and left striatal volume was negatively correlated with CU traits (*p* = .01, svc). However, these findings were not significant when controlling for lifetime/ever CD symptoms (all *p* > .15, svc).

When considering the female CD sample alone, none of the ROIs were negatively or positively correlated with psychopathic or CU traits. However, when we directly compared CD participants with high versus low CU traits, we observed reduced right anterior insula volume in the high CU group (*p* < .05, svc). See Tables S1 and S2 for regions outside the ROIs that were correlated with psychopathic or CU traits at an uncorrected level.

### Correlations with CD symptoms within the female CD group only

None of the ROIs were positively or negatively correlated with lifetime/ever or current CD symptoms. However, given previous research showing reduced dorsolateral prefrontal cortex volume in antisocial populations (Yang & Raine, 2009), it is notable that aggressive CD symptoms were negatively correlated with right dorsolateral prefrontal cortex volume (*z* = 4.83, *p* < .0001, uncorrected; Figure S3). This negative correlation remained when adjusting for ADHD symptoms, psychopathic or CU traits, age, or FSIQ (all *z* ≥ 4.35, *p* < .0001, uncorrected).

### Sex differences in the relationship between CD and brain structure

We used the current dataset and archive data from our previous VBM study on male adolescents (Fairchild et al., 2011) to assess for sex differences in the relationship between CD and brain structure, selecting the 42 male participants who best matched the female sample in age, full-scale IQ, handedness, ethnicity and SES. The male and female CD groups were also matched in lifetime/ever and aggressive CD symptoms and age-of-onset of CD (see Table S3 for participant characteristics). All data were collected using the same scanner, with identical acquisition parameters across participants and intermixed data collection from males and females. These datasets were incorporated into a single DARTEL template using the methods described above.

**Figure 2:**
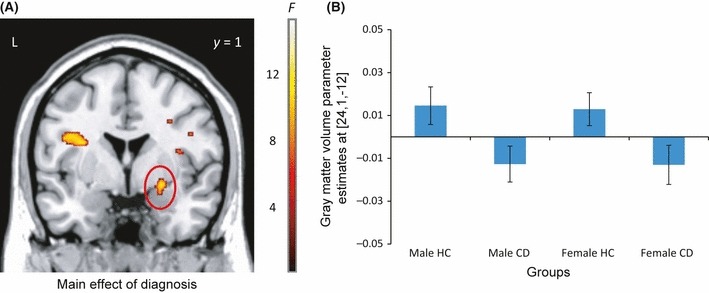
Right amygdala/extended amygdala grey matter volume was reduced in a group of male and female adolescents with CD (*n* = 44), relative to a group of male and female healthy control (HC) subjects (*n* = 40). Panel A shows the amygdala group effect (Montreal Neurological Institute coordinates: *x* = 24, *y* = 1, *z* = −12) in coronal format, whereas panel B displays grey matter volume parameter estimates for the peak voxel in right amygdala for males and females separately. The colour bar represents *F* statistics. The image is thresholded at *p* < .005, uncorrected, for display purposes

**Figure 3:**
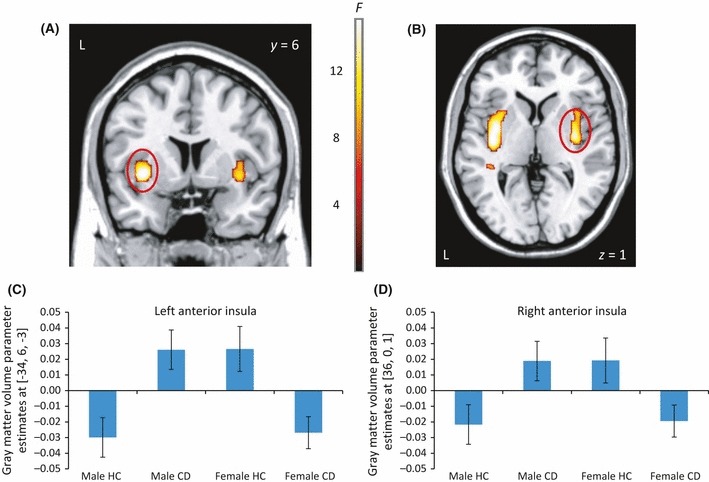
Sex-by-diagnosis interaction in bilateral anterior insula. Panels A and B depict regions that showed significant interactions between sex and diagnosis in coronal and axial format, respectively. The colour bar represents F statistics. The images in A and B are thresholded at *p* < .005, uncorrected, for display purposes. Panels C and D show plots of the interaction for left and right anterior insula, respectively, and provide coordinates of peak voxels

A 2 × 2 ANOVA examining for effects of diagnosis (control vs. CD) and sex (male vs. female) revealed a main effect of diagnosis in the right amygdala/extended amygdala (*p* = .04, svc), reflecting reduced right amygdala volume in the combined CD group relative to controls (Figure 2). This main effect of diagnosis was not qualified by a sex-by-diagnosis interaction, suggesting that male adolescents and female adolescents with CD showed statistically indistinguishable reductions in amygdala volume relative to their respective control groups. We also observed sex-by-diagnosis interactions in left (*p* = .003, svc) and right (*p* = .03, svc) anterior insula (Figure 3), extending into posterior insula on the left side. Underlying these interactions, CD females showed reduced bilateral anterior insula volume (both *p* < .01, svc) relative to control females, whereas CD males showed increased left frontal operculum/insula volume (*p* < .001, uncorrected), compared with control males.

Main effects of sex were observed in bilateral striatum (both *p* < .001, FWE whole-brain correction), bilateral inferior OFC (both *p* ≤ .01, FWE whole-brain correction), right anterior cingulate cortex (*p* = .01, svc) and right amygdala (*p* = .01, svc). Underlying these effects, male adolescents showed increased bilateral striatum and right amygdala volume relative to female adolescents, whereas female adolescents showed increased bilateral OFC and rostral anterior cingulate cortex volume compared with male adolescents.

Table S4 shows coordinates for all main effects and interactions reported above. A similar group effect in right amygdala and sex-by-diagnosis interactions in bilateral anterior insula were obtained when including all 90 male participants from our previous study in the analyses (see Table S5).

## Discussion

To our knowledge, this is the first neuroimaging study to investigate whether brain structure is altered in female adolescents with CD. Consistent with previous studies of male adolescents with CD (Fairchild et al., 2011; Sterzer et al., 2007), we observed reduced grey matter volume in bilateral anterior insula and right striatum in female adolescents with CD relative to healthy controls.

The anterior insula is strongly implicated in empathic processes and, consistent with simulation models of empathy (Preston & de Waal, 2002), is involved in representing physical and affective states of the self (Craig, 2009) and others (Singer et al., 2004). Previous research in male adolescents with CD has reported that anterior insula volume is positively correlated with empathy (Sterzer et al., 2007) and negatively correlated with CD symptoms (Fairchild et al., 2011). Reductions in anterior insula volume may have underpinned the facial emotion recognition impairments previously reported in female adolescents with CD (Fairchild et al., 2010). These impairments were most pronounced for facial expressions of disgust, consistent with research reporting deficits in disgust recognition in patients with insula lesions (Calder, Keane, Manes, Antoun, & Young, 2000; Kipps, Duggins, McCusker, & Calder, 2007).

The reductions in anterior insula volume were robust to statistical adjustment for FSIQ, SES, substance use and psychopathic or CU traits, but were reduced to trends when controlling for ADHD symptoms. This may reflect the high correlation between ADHD and CD symptoms in the current female sample (*r* = .76, *p* < .001). It is worth noting that the anterior insula has not been implicated in previous VBM studies of ADHD (Ellison-Wright, Ellison-Wright, & Bullmore, 2008). We also found that the left anterior insula result remained significant when excluding participants with comorbid ADHD from the analyses, suggesting that our results are at least partly independent of ADHD.

Consistent with our previous findings in male adolescents, we observed reductions in striatal volume in female adolescents with CD. However, these effects were located in right putamen and extending to ventral striatum, rather than the caudate nucleus as in our previous study of male adolescents (Fairchild et al., 2011). The striatum is involved in reward processing and motivational aspects of behaviour (O’Doherty, 2004). Consequently, these structural differences may lead to changes in the processing of motivationally relevant stimuli (Fairchild, 2011). Although the right striatal effects remained significant when controlling for many clinical and personality variables that differed between the groups, these findings were nonsignificant when factoring out ADHD symptoms or excluding participants with comorbid ADHD. This is consistent with a meta-analysis showing that ADHD is associated with reduced right putamen volume (Ellison-Wright et al., 2008).

When investigating for dimensional relationships between CD symptoms and brain volume, we observed a negative correlation between aggressive CD symptoms and right dorsolateral prefrontal cortex (dlPFC) volume. This survived factoring out ADHD symptoms, FSIQ, psychopathic traits, or CU traits. Although not predicted a priori, and therefore requiring replication, this negative correlation is interesting because the right dlPFC is implicated in cognitive control, decision-making and delay of gratification (Baumgartner, Knoch, Hotz, Eisennegger, & Fehr, 2011; Knoch et al., 2006; McClure, Laibson, Loewenstein, & Cohen, 2004). Consequently, reductions in dlPFC volume could affect a range of processes involved in self-control, decision-making and the ability to consider future consequences, thereby increasing risk for (impulsive) aggression. It is also notable that reductions in dlPFC volume have been reported previously in antisocial populations (Yang & Raine, 2009).

When considering the total female sample, we observed a positive correlation between self-reported CU traits and orbitofrontal cortex volume, which remained significant when adjusting for CD or ADHD symptoms. This finding is broadly consistent with a previous study reporting that children with CU traits show increased grey matter concentration in medial orbitofrontal cortex (De Brito et al., 2009). The orbitofrontal cortex is implicated in reward and punishment processing and reversal learning (O’Doherty, 2004; O’Doherty, Kringelbach, Rolls, Hornak, & Andrews, 2001), so this result may explain why previous studies have found alterations in decision-making and reversal learning in adolescents with CD or CU traits (Budhani & Blair, 2005; Fairchild et al., 2009). Of interest, a recent study investigating reward processing in a normative sample found increased medial frontal cortex activation in participants with psychopathic traits (Bjork, Chen, & Hommer, 2012).

### Does the relationship between brain structure and CD differ between sexes?

Contrary to previous research reporting reduced amygdala volume in male adolescents with CD (Fairchild et al., 2011; Huebner et al., 2008; Sterzer et al., 2007), group differences in amygdala volume did not achieve significance in the primary case-control analyses of females. However, additional analyses including archive data from male participants revealed a main effect of diagnosis in right amygdala, reflecting reduced volume in the combined CD group relative to controls. This finding was not qualified by a sex-by-diagnosis interaction, suggesting that male adolescents and female adolescents with CD show statistically indistinguishable reductions in amygdala volume relative to controls (see Figure 2). These results suggest that amygdala abnormalities may contribute to the aetiology of CD in females males, consistent with earlier studies showing impaired fear conditioning in both male adolescents and female adolescents with CD (Fairchild et al., 2008, 2010).

In addition to similarities between male adolescents and female adolescents with CD, we obtained evidence for sex differences in the form of a sex-by-diagnosis interaction in bilateral anterior insula. Female adolescents with CD showed reduced insula volume relative to female controls, whereas male adolescents showed the opposite pattern. The direction of this interaction was unexpected, as we previously reported reduced insula volume in male adolescents with CD (Fairchild et al., 2011). However, it should be noted that the region of insula showing lower volume in our previous study of males was located ventrally (*z* = −18), whereas the present sex-by-diagnosis interaction was detected in a dorsal section of anterior insula (*z* = 1). Furthermore, our previous study also found that male adolescents with CD showed increased volume in left frontal operculum/anterior insula (Fairchild et al., 2011). Future studies could investigate the functional significance of these sex differences in the relationship between CD and anterior insula volume using a combination of structural imaging and behavioural measures assessing interoception and empathy, given the anterior insula’s putative role in these processes (Craig, 2009).

## Limitations

Three limitations are noted. First, the sample size was modest for a VBM study. This reflects the considerable difficulties in recruiting and scanning adolescent girls with CD. Second, our findings should be viewed in the context of high levels of psychiatric comorbidity amongst the female CD participants. While this was not surprising given previous work reporting high rates of MDD and ADHD comorbidity in adolescent girls with CD (Lehto-Salo, Narhi, Ahonen, & Marttunen, 2009), future studies may wish to recruit a larger sample to permit comparisons between ‘pure’ and comorbid CD. Third, the inclusion of a noncomorbid ADHD group would have made it easier to interpret the consequences of controlling for lifetime/ever ADHD symptoms in the CD sample, and allowed us to investigate whether anterior insula volume is reduced in non-comorbid ADHD.

## Conclusions

This study demonstrates that female adolescents with CD show reduced anterior insula and striatal grey matter volume. Our findings also provide preliminary evidence that male adolescents and female adolescents with CD show statistically equivalent reductions in amygdala volume, whereas the relationship between CD and anterior insula volume differs between male adolescents and female adolescents. These results are broadly consistent with those obtained in previous structural imaging studies of male adolescents, and provide novel evidence that female adolescents with CD show structural changes in brain regions implicated in emotion processing and empathy.

## Key points

- Little is known about the neurobiology of CD in female adolescents, because almost all previous neuroimaging studies of conduct disorder (CD) have been restricted to male adolescents alone.
- We used voxel-based morphometry to compare female adolescents with CD and control participants in terms of grey matter volume.
- Female adolescents with CD showed reduced bilateral anterior insula and right striatal grey matter volumes relative to controls.
- When combining the current dataset with archive data obtained from male adolescents, we found a main effect of CD diagnosis in the right amygdala (controls > CD) and sex-by-diagnosis interactions in bilateral anterior insula.
- These results demonstrate that female adolescents with CD show structural changes in brain regions implicated in emotion processing, reward and empathy.

## Supporting information

**Figure S1** Bilateral anterior insula grey mattervolume was reduced in females with adolescence-onset ConductDisorder (*n* = 17) relative to healthy controls.

**Figure S2** Positive correlation between callous-unemotional traits and bilateral orbitofrontal cortex grey matter volume.

**Figure S3** Negative correlation between aggressive Conduct Disorder symptoms and right dorsolateral prefrontal cortex grey matter volume.

**Table S1** Correlations between psychopathic or callous-unemotional traits and grey matter volume in the total female sample

**Table S2** Correlations between psychopathic or callous-unemotional traits and grey matter volume in the female Conduct Disorder group alone

**Table S3** Characteristics of the male and female participants included in the analyses testing for sex differences

**Table S4** Coordinates and cluster sizes for theMain Effects of Diagnosis and Sex, and Sex × Diagnosis interactions for the voxel-based morphometry analyses testing for sex differences with matched male and female groups

**Table S5** Coordinates and cluster sizes for theMain Effects of Diagnosis and Sex, and Sex × Diagnosis interactions for the voxel-based morphometry analyses testing for sex differences with 90 male and 42 female subjec
