# Supplementary material for: Brain structure abnormalities in adolescent girls with conduct disorder
Source: J Child Psychol Psychiatry. 2012 Oct 22;54(1):86–95. doi: 10.1111/j.1469-7610.2012.02617.x (PMC3562487; doi:10.1111/j.1469-7610.2012.02617.x)
Supplement: Supplementary file 1 [file jcpp0054-0086-SD1.pptx]

## Slide 1
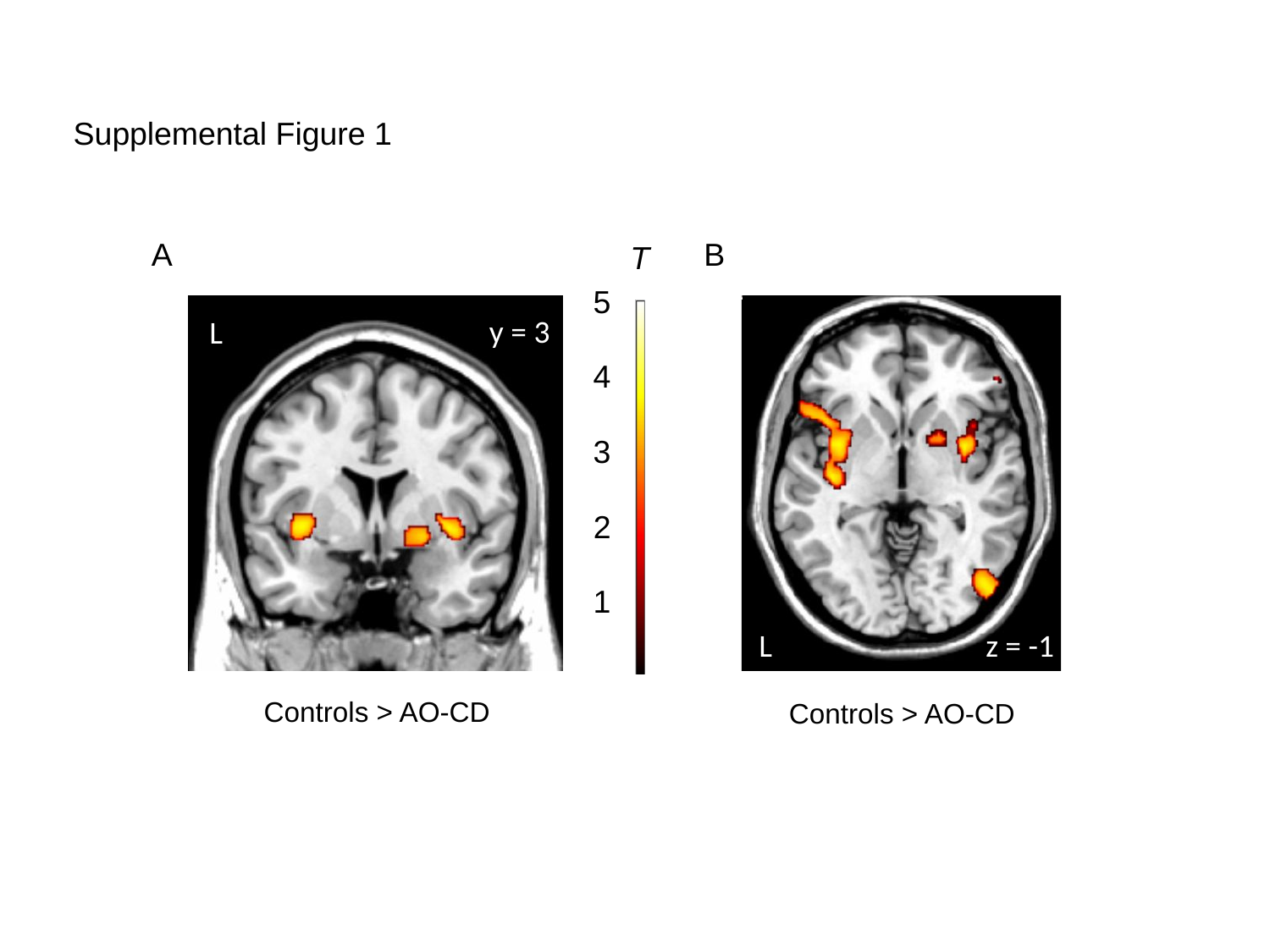

Supplemental Figure 1
A
B
T
5
y = 3
L
4
3
2
1
L
z = -1
Controls > AO-CD
Controls > AO-CD

## Slide 2
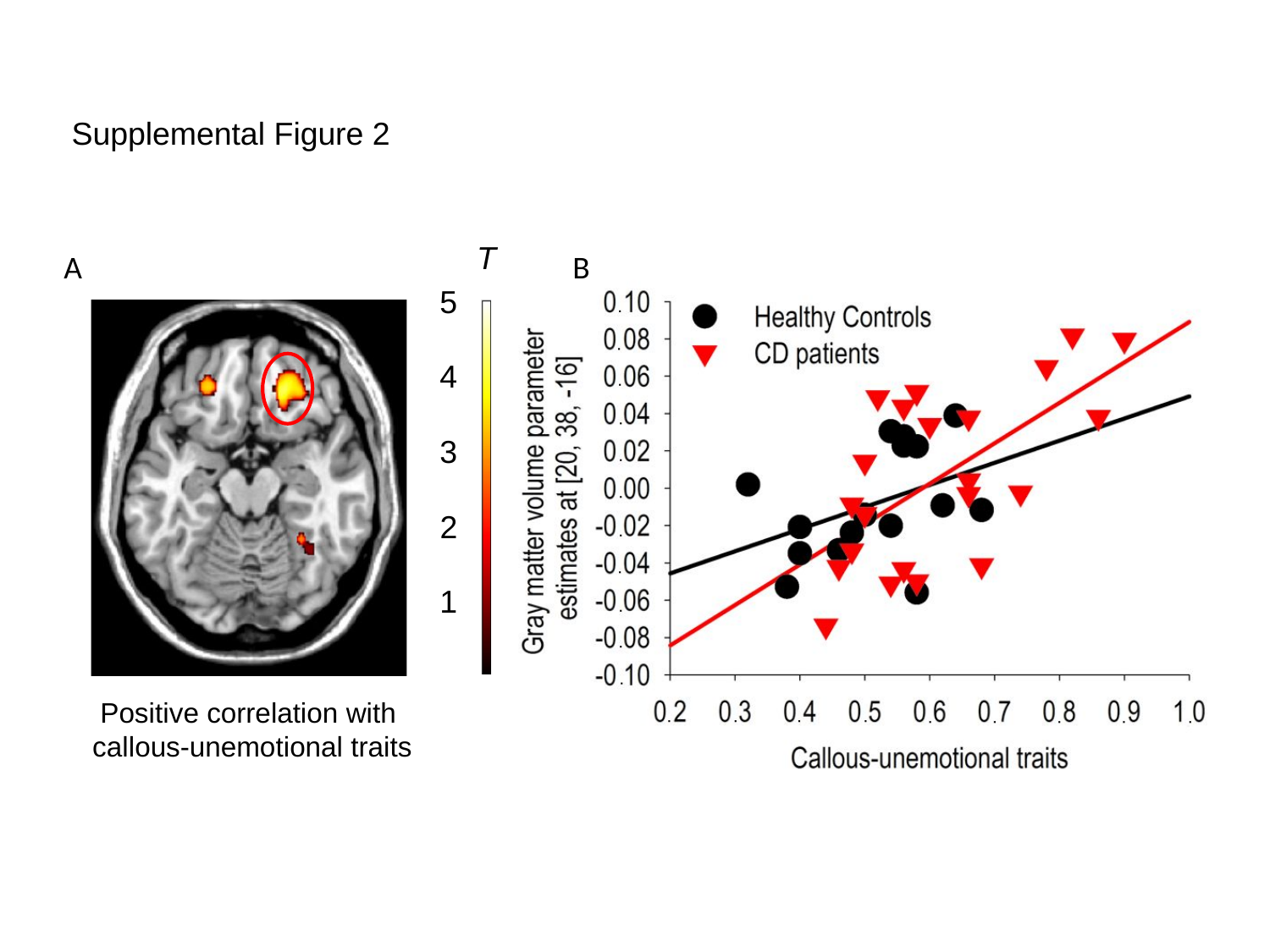

Supplemental Figure 2
T
5
4
3
2
1
A
B
L
z = -16
Positive correlation with
 callous-unemotional traits

## Slide 3
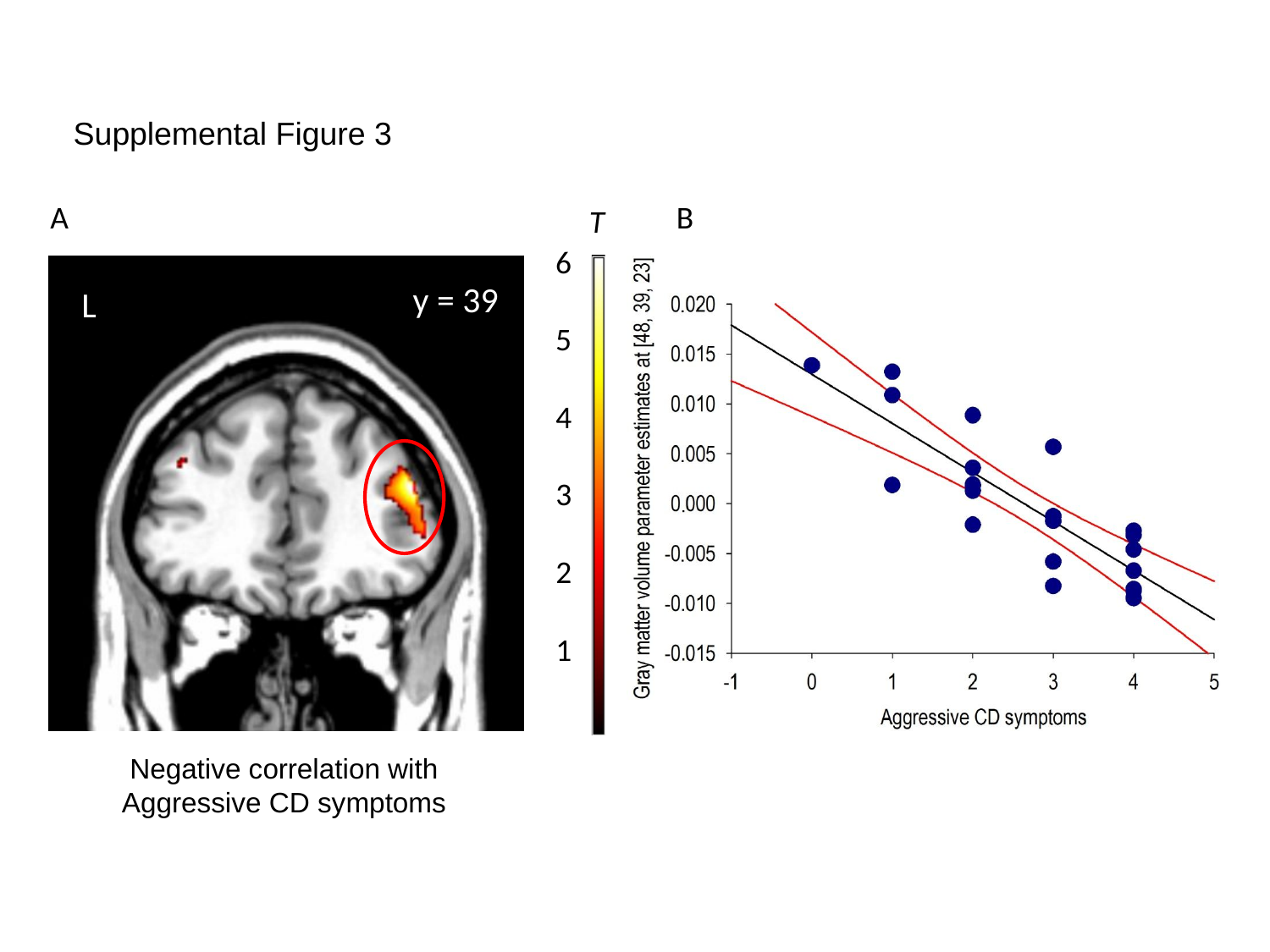

Supplemental Figure 3
B
A
T
6
5
4
3
2
1
x = 48
y = 39
L
Negative correlation with
Aggressive CD symptoms
