# Supplementary material for: Brain structure abnormalities in adolescent girls with conduct disorder
Source: J Child Psychol Psychiatry. 2012 Oct 22;54(1):86–95. doi: 10.1111/j.1469-7610.2012.02617.x (PMC3562487; doi:10.1111/j.1469-7610.2012.02617.x)
Supplement: Supplementary file 2 [file jcpp0054-0086-SD2.docx]

**Supplemental Table 1: Correlations between psychopathic or callous-unemotional traits and grey matter volume in the total female sample**

| Cerebral regions | Hemisphere | Local Maxima, Z | No. of significant  voxels in cluster | MNI Coordinates | | |
| --- | --- | --- | --- | --- | --- | --- |
| *Positive correlations with YPI Total* |  |  |  | X | Y | Z |
| Superior occipital cortex | L | 3.76 | 60 | -18 | -79 | 24 |
| Medial frontal gyrus | R | 3.59 | 97 | 22 | 39 | 28 |
| Cuneus | L | 3.59 | 28 | -22 | -70 | 12 |
| *Negative correlations with YPI Total* |  |  |  |  |  |  |
| Striatum | L | 3.89^a^ | 544 | -32 | 3 | 3 |
| Anterior insula | L | 3.59^a^ | same cluster as above | -36 | 5 | 1 |
|  | R | 3.46^a^ | 35 | 39 | 15 | 10 |
| Inferior frontal gyrus | R | 3.44 | 13 | 39 | 27 | 6 |
| Striatum | R | 3.38 | 28 | 24 | 12 | 9 |
|  | R | 3.14 | 19 | 22 | 18 | -3 |
| Insula, sub-lobar | R | 3.28 | 29 | 39 | 6 | -12 |
| *Positive correlations with YPI CU traits* |  |  |  |  |  |  |
| Superior orbitofrontal cortex | R | 3.81^a^ | 366 | 20 | 38 | -17 |
| Middle orbitofrontal cortex | L | 3.47^b^ | 27 | -24 | 39 | -15 |
| Superior occipital cortex | L | 3.61 | 66 | -20 | -81 | 25 |
| *Negative correlations with YPI CU traits* |  |  |  |  |  |  |
| Striatum | L | 4.26^a^ | 416 | -24 | 0 | 15 |
| Anterior insula | L | 3.30^a^ | same cluster as above | -33 | 5 | -3 |
| Anterior insula | R | 3.95^a^ | 121 | 39 | 16 | 12 |
| Striatum | R | 3.55 | 43 | 24 | 9 | 12 |
|  | R | 3.40 | 46 | 27 | 12 | -6 |
| Globus pallidus | R | 3.24 | 18 | 21 | -4 | 1 |
| Cingulate gyrus | R | 3.97 | 349 | 20 | 6 | 48 |
| Precuneus | R | 3.70 | 33 | 14 | -60 | 49 |

Abbreviations: MNI, Montreal Neurological Institute; YPI CU traits, Youth Psychopathic traits Inventory callous-unemotional traits subscale (self-report); YPI Total, Youth Psychopathic traits Inventory Total score (self-report).

^a^ p < 0.05, Family-Wise Error (small-volume correction); ^b^ p = 0.05, Family-Wise Error (small-volume correction)

Correlations in all other regions met the criteria of p < 0.001, uncorrected, for ≥ 10 contiguous voxels

**Supplemental Table 2: Correlations between psychopathic or callous-unemotional traits and grey matter volume in the female Conduct Disorder group alone**

| Cerebral regions | Hemisphere | Local Maxima, Z | No. of significant  voxels in cluster | MNI Coordinates | | |
| --- | --- | --- | --- | --- | --- | --- |
| *Positive correlations with YPI Total* |  |  |  | X | Y | Z |
| Superior frontal gyrus | R | 3.61 | 116 | 26 | 42 | 36 |
| Fusiform/lingual gyrus | R | 3.27 | 14 | 24 | -84 | -8 |
| *Negative correlations with YPI Total* |  |  |  |  |  |  |
| Precuneus | R | 4.06 | 141 | 20 | -46 | 33 |
| Posterior cingulate cortex | R | 3.85 | 113 | 10 | -51 | 19 |
| Precuneus | R | 3.82 | 86 | 9 | -60 | 46 |
| Inferior parietal lobule | L | 3.65 | 37 | -38 | -34 | 27 |
| Posterior insula | R | 3.61 | 85 | 45 | -27 | 22 |
| Precuneus | R | 3.32 | 10 | 34 | -66 | 36 |
| Posterior insula | L | 3.25 | 11 | -50 | -39 | 19 |
| Precuneus | L | 3.20 | 35 | -4 | -55 | 42 |
| *Positive correlations with YPI CU traits* |  |  |  |  |  |  |
| Fusiform/lingual gyrus | R | 4.05 | 182 | 26 | -82 | -6 |
| Occipital cortex, subgyral | R | 3.60 | 68 | 33 | -58 | -11 |
| *Negative correlations with YPI CU traits* |  |  |  |  |  |  |
| Precuneus | R | 4.17 | 166 | 9 | -60 | 46 |
|  | R | 3.79 | 88 | 10 | -49 | 18 |
|  | R | 3.35 | 26 | 34 | -66 | 34 |
| Posterior cingulate cortex | R | 3.29 | Same cluster as above | 12 | -46 | 19 |
| Cingulate gyrus | R | 3.61 | 47 | 20 | -40 | 34 |
| Posterior insula | R | 3.51 | 96 | 42 | -25 | 25 |
| Parahippocampal gyrus | R | 3.33 | 14 | 18 | -42 | -3 |
| Parietal cortex | L | 3.31 | 14 | -36 | -34 | 25 |

Abbreviations: MNI, Montreal Neurological Institute; YPI CU traits, Youth Psychopathic traits Inventory callous-unemotional traits subscale (self-report); YPI Total, Youth Psychopathic traits Inventory Total score (self-report).

Correlations in all regions met the criteria of p<0.001, uncorrected, for ≥ 10 contiguous voxels

**Supplemental Table 3: Characteristics of the male and female participants included in the analyses testing for sex differences**

| **Measure** | **Male HC**  **(n = 20)** | | **Male CD**  **(n = 22)** | | **Female HC**  **(n = 20)** | | **Female CD**  **(n = 22)** | | P values |
| --- | --- | --- | --- | --- | --- | --- | --- | --- | --- |
|  | **Mean** | **SD** | **Mean** | **SD** | **Mean** | **SD** | **Mean** | **SD** |  |
| **Age (years)**  **Full-Scale IQ**  **YPI total score**  **YPI CU subscale**  **Lifetime CD symptoms**  **Current CD symptoms**  **Aggressive CD symptoms**  **Lifetime ADHD symptoms**  **Current ADHD symptoms** | 18.03  102.1  1.97  0.59  0.40  0.05  0.10  2.36  1.37 | 0.66  9.13  0.30  0.11  0.68  0.22  0.31  2.03  1.71 | 17.38  103.09  2.45  0.72  7.18  5.09  2.82  5.75  3.50 | 1.03  9.11  0.40  0.12  2.34  2.33  1.26  3.68  3.67 | 17.55  105.80  1.59  0.52  0.38  0.12  0.06  1.95  1.60 | 0.67  9.52  0.31  0.10  0.62  0.34  0.25  2.16  1.85 | 17.23  99.77  2.07  0.62  7.59  2.73  2.64  8.18  6.00 | 1.67  7.90  0.42  0.13  2.26  2.53  1.22  3.70  3.34 | S=0.13, D=0.08  S=0.91, D=0.20  S<0.01, D<0.01  S<0.01, D<0.01  S=0.63, D<0.01  S<0.01, D<0.01,*  S=0.61, D<0.01  S=0.15, D<0.01,*  S=0.03, D<0.01 |
|  | **N** | **%** | **N** | **%** | **N** | **%** | **N** | **%** |  |
| *ACORN socioeconomic*  *status*  Wealthy achievers (1)  Urban prosperity (2)  Comfortably off (3)  Moderate means (4)  Hard-pressed (5)  *Ethnicity:*  Caucasian  Non-white | 2  5  5  2  6  19  1 | 10.0  25.0  25.0  10.0  30.0  95.0  5.0 | 1  6  6  2  7  20  2 | 4.5  27.3  27.3  9.1  31.8  90.9  9.1 | 9  0  6  0  5  20 | 45.0  0.0  30.0  0.0  25.0  100.0 | 4  5  4  1  8  21  1 | 18.2  22.7  18.2  4.5  36.4  95.5  4.5 | Exact test=0.07  Exact test=0.90 |

Key: ADHD, Attention Deficit/Hyperactivity Disorder; HC, healthy control; CD, Conduct Disorder; CU, callous-unemotional; EO/EO-CD, childhood-onset Conduct Disorder; IQ, intelligence quotient; YPI, Youth Psychopathic traits Inventory. In the P value column, S indicates the significance value for the main effect of sex, D indicates the main effect of Diagnosis, and * denotes a significant sex-by-diagnosis interaction (p<0.05).

**Supplemental Table 4: Coordinates and cluster sizes for the Main Effects of Diagnosis and Sex, and Sex × Diagnosis interactions for the voxel-based morphometry analyses testing for sex differences with matched male and female groups**

| Cerebral regions | Hemisphere | Local Maxima, F | No. of significant  voxels in cluster | MNI Coordinates | | |
| --- | --- | --- | --- | --- | --- | --- |
| *Main Effects of Diagnosis* |  |  |  | X | Y | Z |
| Striatum | R | 12.95 | 59 | 22 | 6 | -9 |
| Amygdala | R | 9.64^b^ | same cluster as above | 24 | 2 | -12 |
| Middle temporal gyrus | R | 18.64 | 155 | 51 | -12 | -24 |
| Middle occipital gyrus | L | 13.48 | 68 | -40 | -91 | 7 |
| *Interaction between Sex and Diagnosis* |  |  |  |  |  |  |
| Anterior insula | L | 19.10^b^ | 887 | -34 | 6 | -3 |
| Posterior insula | L | 18.11 | same cluster as above | -39 | -13 | 3 |
| Posterior insula | R | 15.09 | 144 | 35 | -9 | 3 |
| Anterior insula | R | 12.60^b^ | same cluster as above | 36 | 0 | 1 |
| Superior occipital cortex | L | 19.09 | 263 | -18 | -88 | 27 |
| *Main Effects of Sex* |  |  |  |  |  |  |
| Striatum | L | 45.73^a^ | 2233 | -22 | -9 | 1 |
|  | R | 51.18^a^ | 2538 | 22 | -6 | 3 |
| Superior orbitofrontal cortex | L | 40.01^a^ | 2012 | -18 | 27 | -12 |
|  | R | 31.30^a^ | 2389 | 16 | 29 | -14 |
| Cerebellum | L | 29.05^a^ | 698 | -54 | -64 | -24 |
|  | R | 37.59^a^ | 4600 | 56 | -61 | -26 |
| Superior temporal gyrus | L | 29.34^a^ | 302 | -51 | 21 | -17 |
|  | R | 30.11^a^ | 77 | 56 | 18 | -8 |
| Midbrain | Medial | 27.46^a^ | 563 | -2 | -36 | -12 |
| Insula | L | 16.01^b^ | 104 | -28 | 29 | 1 |
| Amygdala | R | 13.46^b^ | 17 | 30 | 0 | -18 |

^a^ p<0.05, Family-Wise Error (whole-brain correction); ^b^ p<0.05, Family-Wise Error (small-volume correction).

All other effects reported were significant at p<0.001, uncorrected, for ≥10 contiguous voxels; MNI, Montreal Neurological Institute

**Supplemental Table 5: Coordinates and cluster sizes for the Main Effects of Diagnosis and Sex, and Sex × Diagnosis interactions for the voxel-based morphometry analyses testing for sex differences with 90 male and 42 female subjects**

| Cerebral regions | Hemisphere | Local Maxima, F | No. of significant  voxels in cluster | MNI Coordinates | | |
| --- | --- | --- | --- | --- | --- | --- |
| *Main Effects of Diagnosis* |  |  |  | X | Y | Z |
| Putamen | R | 18.45^b^ | 447 | 22 | 6 | -9 |
| Amygdala | R | 14.51^b^ | same cluster as above | 30 | 4 | -20 |
| Anterior insula | R | 12.59^b^ | 24 | 38 | 4 | -3 |
| Inferior temporal gyrus | R | 25.94 | 321 | 50 | -13 | -24 |
| Middle frontal gyrus | R | 12.78 | 15 | 50 | 8 | 37 |
| *Interaction between Sex and Diagnosis* |  |  |  |  |  |  |
| Anterior insula | L | 15.91^b^ | 321 | -36 | 5 | -2 |
| Posterior insula | L | 13.81 | same cluster as above | -36 | -1 | 1 |
| Anterior insula | R | 12.62^b^ | 36 | 38 | 12 | -6 |
| Caudate nucleus | L | 15.08^b^ | 157 | -15 | -16 | 19 |
| Precuneus | L | 18.13 | 151 | -18 | -79 | 27 |
| *Main Effects of Sex* |  |  |  |  |  |  |
| Putamen/striatum | L | 55.76^a^ | 2989 | -24 | 6 | 6 |
|  | R | 63.64^a^ | 3262 | 24 | 2 | 4 |
| Amygdala | R | 19.00^b^ | same cluster as above | 30 | 0 | -20 |
| Inferior orbitofrontal cortex | L | 37.40^a^ | 4855 | -33 | 38 | -11 |
|  | R | 28.86^a^ | same cluster as above | 32 | 32 | -11 |
| Rostral anterior cingulate | R | 21.38^b^ | same cluster as above | 10 | 32 | -11 |
| Fusiform gyrus | L | 35.20^a^ | 781 | -50 | -63 | -24 |
|  | R | 44.88^a^ | 3431 | 54 | -63 | -24 |
| Midbrain | Medial | 30.09^a^ | 823 | -2 | -36 | -12 |
| Cingulate gyrus | R | 25.80^a^ | 653 | 6 | -36 | 39 |
| Parietal cortex | R | 25.67^a^ | 421 | 20 | -37 | 60 |
| Superior temporal gyrus | L | 24.29^a^ | 139 | -50 | 20 | -15 |
|  | R | 24.46^a^ | 45 | 54 | 18 | -8 |

^a^ p<0.05, Family-Wise Error (whole-brain correction); ^b^ p<0.05, Family-Wise Error (small-volume correction).

All other effects reported were significant at p<0.001, uncorrected, for ≥10 contiguous voxels; MNI, Montreal Neurological Institute
